# Immunomodulatory potential of rapamycin-loaded mesoporous silica nanoparticles: pore size-dependent drug loading, release, and in vitro cellular responses

**DOI:** 10.1007/s13346-024-01575-0

**Published:** 2024-04-01

**Authors:** Ana M. Pérez-Moreno, Carlos J. Aranda, María José Torres, Cristobalina Mayorga, Juan L. Paris

**Affiliations:** 1 Allergy Research Group, Instituto de Investigación Biomédica de Málaga y Plataforma en Nanomedicina- IBIMA Plataforma BIONAND. RICORS “Enfermedades inflamatorias”, Málaga, Spain; 2 Allergy Unit, Hospital Regional Universitario de Málaga-HRUM, Málaga, Spain; 3 Departamento de Medicina y Dermatología, Universidad de Málaga, Málaga, España

**Keywords:** Mesoporous silica nanoparticles, Dendritic cells, Immunomodulation, Nanomedicine, Rapamycin

## Abstract

Rapamycin is a potent immunosuppressive drug that has been recently proposed for a wide range of applications beyond its current clinical use. For some of these proposed applications, encapsulation in nanoparticles is key to ensure therapeutic efficacy and safety. In this work, we evaluate the effect of pore size on mesoporous silica nanoparticles (MSN) as rapamycin nanocarriers. The successful preparation of MSN with 4 different pore sizes was confirmed by dynamic light scattering, zeta potential, transmission electron microscopy and N_2_ adsorption. In these materials, rapamycin loading was pore size-dependent, with smaller pore MSN exhibiting greater loading capacity. Release studies showed sustained drug release from all MSN types, with larger pore MSN presenting faster release kinetics. In vitro experiments using the murine dendritic cell (DC) line model DC2.4 showed that pore size influenced the biological performance of MSN. MSN with smaller pore sizes presented larger nanoparticle uptake by DC2.4 cells, but were also associated with slightly larger cytotoxicity. Further evaluation of DC2.4 cells incubated with rapamycin-loaded MSN also demonstrated a significant effect of MSN pore size on their immunological response. Notably, the combination of rapamycin-loaded MSN with an inflammatory stimulus (lipopolysaccharide, LPS) led to changes in the expression of DC activation markers (CD40 and CD83) and in the production of the proinflammatory cytokine TNF-α compared to LPS-treated DC without nanoparticles. Smaller-pored MSN induced more substantial reductions in CD40 expression while eliciting increased CD83 expression, indicating potential immunomodulatory effects. These findings highlight the critical role of MSN pore size in modulating rapamycin loading, release kinetics, cellular uptake, and subsequent immunomodulatory responses.

**Graphical abstract:**

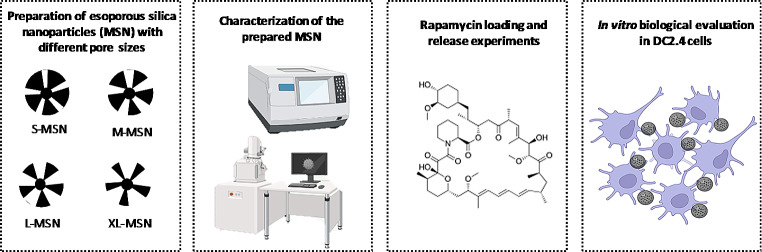

**Supplementary Information:**

The online version contains supplementary material available at 10.1007/s13346-024-01575-0.

## Introduction

Rapamycin is a potent immunosuppressive agent used in the clinic to prevent immune rejection after an organ transplant [1]. Even though the drug is currently used for this immunosuppressive activity, the mammalian target of rapamycin (mTOR) is associated with a multitude of diseases, such as cancer, diabetes, and neurological diseases [1, 2], so many other therapeutic applications have been proposed in recent years, such as in combination cancer chemotherapy [2, 3], as an anti-aging drug [4] and as an adjuvant in antigen-specific immune tolerance generation [5–9]. For many of these applications, the encapsulation of the drug within a nanocarrier becomes critical to ensure therapeutic efficacy, in order to direct the drug towards specific target cells or even just to reduce the systemic dose of the drug (to prevent generalized immunosuppression). Thus, different nanoparticle carriers have been employed to deliver rapamycin, such as lipidic [10] or polymeric nanoparticles [11–16]. In the context of immunomodulatory drug nanocarriers, silica nanoparticles can be particularly interesting, as previous works have identified their potential adjuvant role that could be added to the effect of the drug being carried [17]. Mesoporous silica nanoparticles (MSN), in particular, present pores in the range of 2 to 50 nm that can be used to carry large amounts of therapeutics [18, 19]. Regarding the adjuvant properties of MSN, several authors have reported that variations in MSN pore size have implications for their adjuvant potential. For example, Vallhov et al. reported that MSN pore size determined their effect on human dendritic cells (DCs) in vitro [20]. Furthermore, Wang et al. also reported that the in vitro immunogenicity of pathogen-associated molecular patterns (PAMPs)-modified MSN grew as pore size was increased [21]. Moreover, in that same work, the authors demonstrated that the in vivo immunogenicity of PAMPs-modified large-pore MSN was larger than that of the common adjuvant Alum. Besides its effect on the immunogenicity of MSN, tuning the pore size within this mesoporous range has been reported to be critically important for cargo loading and release not only of macromolecules [22, 23], but also of small molecule drugs [24–28]. With regards to the effect of pore size of MSN loaded with small molecules (as is the drug chosen for this work, rapamycin, with an estimated molecular size of 1.5-2 nm [29]), prior reports have mainly focused on drugs that are poorly soluble in aqueous media (which is also the case for rapamycin), such as doxorubicin [30], paclitaxel [28], metoprolol [26], nimesulide [25] or ibuprofen [27]. Encapsulation of these poorly soluble drugs within nanoparticles enhances their solubility through sustained release from MSN, and particles with larger pore size generally release larger amounts of the cargo in a shorter time, which enables selecting a formulation of a particular pore size that provides the desired release kinetics. While some examples of mesoporous materials loaded with rapamycin have been previously reported [3, 29, 31, 32], no systematic evaluation of mesopore size on rapamycin loading, release and biological effect have been produced. In this work, we report the preparation and characterization of rapamycin-loaded MSN of 4 different pore sizes (in the range 3–12 nm). As rapamycin is an immunosuppressive drug, the biological effect of the loaded nanocarriers was evaluated in vitro using an established model for DCs, the murine cell line DC2.4.

## Materials and methods

### Materials

The following reagents were purchased from Merck (Sigma‒Aldrich, Spain) and were used without further purification: tetraethylorthosilicate (TEOS), cyclohexane, triethanolamine, cetyltrimethylammonium chloride (CTAC), ammonium nitrate, ethanol, hydrochloric acid, rhodamine B isothiocyanate (RITC), aminopropyltriethoxysilane (APTES), phosphate buffered saline (PBS) tablets, Roswell Park Memorial Institute (RPMI)-1640 culture medium, fetal bovine serum (FBS), nonessential amino acids, L-glutamine, β-mercaptoethanol and cell proliferation reagent WST-1. Rapamycin was purchased from Alfa Aesar (USA). DC2.4 murine cell line was also obtained from Merck (Sigma‒Aldrich, Spain) and was cultured following the manufacturer’s instructions. ELISA kit (ELISA Flex brand) for the determination of TNF-α was obtained from Mabtech (Sweden). FITC anti-mouse CD40 and PE/Cy7 anti-mouse CD83 antibodies for flow cytometry was purchased from Biolegend (USA).

### Characterization techniques

Dynamic light scattering (DLS) and Z-potential measurements were performed with a Malvern Zetasizer Nano ZS90 instrument, checking both particle size and surface charge. DLS and Z potential measurements were performed in water using either pristine MSN or MSN which had been previously incubated for 1 h in phenol red-free RPMI-1640 culture medium supplemented with 10% FBS. The instrument used was equipped with a “red laser” (ʎ = 300 nm), and DLS measurements were performed with a detection angle of 90°, while the Smoluchowski approximation was used for Z-potential measurements. To check the morphology and the different pore sizes of the nanoparticles, the characterization of the nanoparticles was performed by transmission electron microscopy (TEM) on a Thermo Fisher Scientific Tecnai G2 20 Twin using copper grids of mesh size 200 coated with a Formvar-Carbon film. Nitrogen adsorption (in a Micromeritics ASAP 2020 unit) measurements were carried out at the Central Research Support Services (SCAI) of the University of Malaga (UMA). UV‒Vis spectrophotometry was carried out using an Epoch plate reader (Agilent BioTek, USA). Confocal microscopy was performed using a Leica SP5 HyD Confocal Microscope (Leica, Germany). Flow cytometry was carried out in a CytoFLEX cytometer (Beckman Coulter, USA).

### Synthesis of MSN

MSN of varying mesopore sizes were prepared as previously described [33]. In this method, the condensation of TEOS takes place in a biphasic water/cyclohexane system, using triethanolamine as the base and CTAC as the structure-directing agent. The aqueous phase was a mixture of 24 mL of a commercial aqueous solution of CTAC (25% w/v)), 0.18 g of triethanolamine and 36 mL of deionized water. The organic phase was made of 20 mL of a mixture of cyclohexane with TEOS. The concentration of TEOS varied depending on the desired pore size: 40% for S-MSNs, 20% for M-MSNs, 10% for L-MSNs and 5% for XL-MSNs. The synthesis reaction was carried out at 50 °C for 24 h. Then, the surfactant was extracted by ion exchange with an ethanolic solution of ammonium nitrate (10 mg/mL) at reflux for 1 h, followed by a second reflux for 2 h in an ethanolic solution of 12 mM HCl. Finally, the materials were washed with ethanol 3 times to afford the final materials, which were dried and stored at room temperature until further use. Fluorescent MSNs were also obtained by adding a mixture of 1.5 mg of RITC and 15 µL of APTES in 1 mL of ethanol in the aqueous phase during MSN synthesis.

### Rapamycin loading and release from MSN

Rapamycin was loaded in MSNs by dispersing 10 mg of MSNs in a 10 mg/mL solution of the cargo in absolute ethanol and stirring overnight. Then, the loaded particles were collected by centrifugation (7,000 g for 10 min), dried and stored at -20 °C until further use. Non-loaded cargo was quantified from the supernatant by UV‒Vis spectrophotometry (λ_ABS_ = 278 nm). For release experiments, loaded particles were suspended in PBS and stirred at 37 °C. At different time points, the particles were centrifuged, released cargo was quantified by UV‒Vis spectrophotometry (λ_ABS_ = 278 nm), and the particles were resuspended in fresh PBS to continue stirring at 37 °C.

### In vitro evaluation of MSN in a model of dendritic cells

A mouse DCs line (DC 2.4) was used to evaluate the immunological effect of MSNs [34, 35]. DC2.4 cells were cultured following the manufacturer’s instructions in RPMI 1640 culture medium supplemented with 10% fetal bovine serum, nonessential amino acids, L-glutamine and β-mercaptoethanol. Cells were grown in an incubator at 37 °C with 5% CO_2_. The day prior to the experiment, DC2.4 cells were seeded in a 96 well plate (50,000 cells per well). For cellular uptake experiments, DC 2.4 cells were incubated with RITC-labelled MSNs for 2 h at a concentration of 5, 10 or 20 µg/mL in complete medium. Then, non-internalized nanoparticles were removed by washing the cells with PBS and adding fresh complete medium. Twenty-four hours later, MSN uptake was evaluated by flow cytometry and confocal microscopy. For confocal microscopy, µ-Slide 8 Well (purchased from ibidi, USA) were used. These cells were fixed with 2% paraformaldehyde in PBS for 5 min, followed by permeabilization with 0.1% Triton X-100, and staining the cytoplasm with Phalloidin-Atto 488 and the nuclei with DAPI. Stained cells were kept in PBS until evaluation by confocal microscopy.

To evaluate the biological effect, DC2.4 cells were incubated with empty and rapamycin-loaded nanoparticles (nonlabelled) as described for uptake experiments, and 24 h later the cells were collected by trypsinization and stained with fluorescent antibodies for different membrane DC markers (CD40, CD83) whose expression was assessed by flow cytometry after cell fixation. Two different experiments were performed: either using a constant MSN concentration of 10 µg/mL or a constant rapamycin concentration of 1 µg/mL. For free rapamycin and lipopolysaccharide (LPS), 1 µg/mL and 100 ng/mL were used, respectively. The levels of the pro-inflammatory cytokine TNF-α was also evaluated by ELISA in the supernatants of DC2.4 cells incubated with the nanoparticles, following the manufacturer’s instructions.

## Results and discussion

**Fig. 1:**
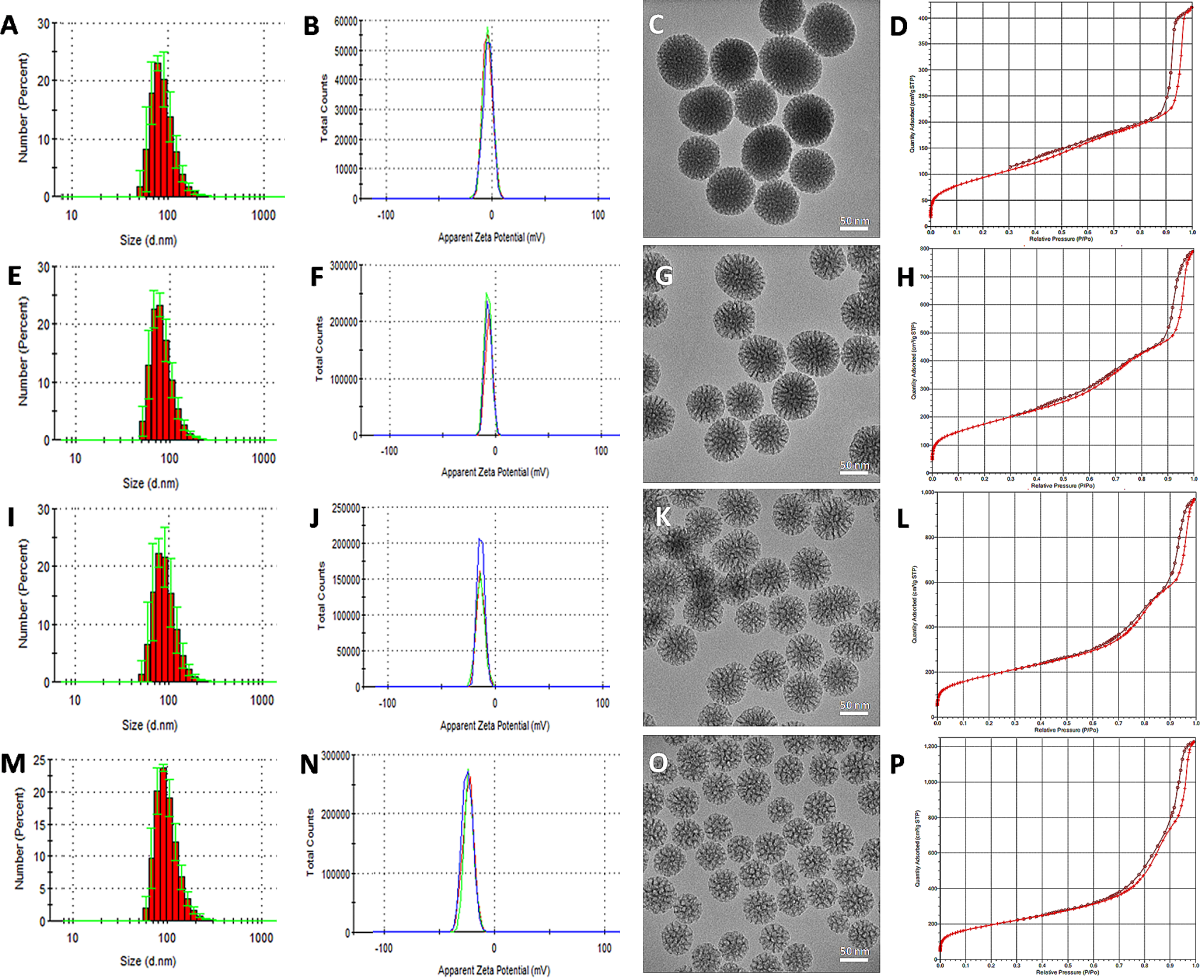
Characterization of the prepared MSN: S-MSN **(A-D)**, M-MSN **(E-H)**, L-MSN **(I-L)** and XL-MSN **(M-P)**. Nanoparticle size histograms determined by DLS **(A,E,I,M)**; Apparent zeta potential distribution **(B,F,J,N)**; TEM micrographs showing nanoparticle morphology and porosity **(C,G,K,O)**; N_2_ adsorption isotherms **(D,H,L,P)**.

The successful preparation of the desired MSN with different pore sizes was confirmed by different characterization techniques (Fig. 1, Table S1). All different MSN presented a peak hydrodynamic diameter between 78.82 and 91.28 nm and slightly negative zeta potential (between − 4.09 and − 23.8 mV). TEM micrographs also confirmed the round morphology of the particles and the presence of mesopores of different sizes (Fig. 1). Finally, the textural properties of the MSN were evaluated by N_2_ adsorption, showing in all cases a large surface area (between 339.3 and 695.3 m^2^/g) and pore diameters of 3.19 nm (S-MSN), 5.55 nm (M-MSN), 8.39 nm (L-MSN) and 11.3 nm (XL-MSN) for the different types of nanoparticles (Fig. 1, Table S1). These results are in good agreement with previous reports of MSN prepared by the same biphasic method, with similar particle sizes and textural properties [24, 33, 35, 36]. However, it should be noted that when evaluating the biological performance of nanoparticles in vitro, it is important to also assess the characteristics of the nanoparticles in suspension in the culture medium to be used for the biological evaluation, as these properties might be different in this medium, for example if aggregation takes place or if the characteristics of the formed protein corona are very different between the different nanoparticles being tested. For this reason, we carried out DLS and Z potential measurements of the different nanoparticles after incubation in culture medium (with 10% FBS) for 1 h. The results (Figure S1, Table S1) show that there were only small increases in particle size (with all measured hydrodynamic diameters in the range of 90–125 nm), ruling out any large-scale aggregation caused by incubation in culture medium with serum. Furthermore, the Z potential values obtained for all MSN types were very similar (in the range of -24.1 to -26.1 mV), with no significant differences between the particles of different pore sizes. This was in contrast with the Z potential values of the pristine MSN in water, which presented a much broader range of Z potential values. This change in Z potential was most likely caused by the formation of a protein corona surrounding all MSN types that produced these more homogeneous surface charge characteristics. It is worth noting that having similar Z potential values does not necessarily imply that the proteins surrounding the different MSN types are the same or are present in the same proportions, and previous reports have shown that differences in MSN characteristics, such as surface chemistry [37], morphology [38] and pore size [39] have a large impact on protein corona formation and composition. Thus, as in culture medium there were no relevant differences in size and Z potential among the different MSN formulations, potential differences in their in vitro biological performance will be due to either their different textural properties or differences in the composition of their protein corona.

**Fig. 2:**
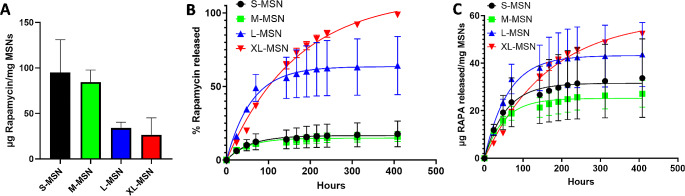
Rapamycin loading **(A)** and release **(B, C)** in MSN of different pore sizes. Data are Mean ± SD, *n* = 3

Rapamycin was loaded in MSN with different pore sizes, observing a maximum loading for S-MSN with a clear decrease in drug loaded as the mesopore size was increased (Fig. 2A). This result was expected, as rapamycin is a hydrophobic small molecule drug which will be best retained in mesopores slightly above its molecular size. In previous reports with larger cargo molecules, loading was increased in MSN of larger pore sizes [28, 35]. Thus, our results in context with previous literature corroborate that the interaction between mesopore size and cargo molecular weight determines which is the optimal MSN formulation to load each cargo molecule. Release experiments showed that the rapamycin release was faster for XL-MSN and L-MSN compared to M-MSN and S-MSN (Fig. 2B). This behavior can be explained by the larger accessibility of the solvent in MSN with larger pores, which drives a faster release from the nanoparticles. Furthermore, this result is in good agreement with previous reports which showed larger and faster release of different poorly soluble small molecule drugs when MSN pore size was increased [25–28]. However, as cargo loading was much larger in MSN with smaller pores, there is an interplay between drug loading and release when comparing the absolute drug mass being released from MSN (Fig. 2C). Thus, even though at shorter time points the amount of rapamycin released from all formulations is relatively similar (or even smaller for XL-MSN), at longer time points the amount of rapamycin released from L-MSN and especially from XL-MSN is much larger than that released from the same mass of S-MSN or M-MSN particles. In any case, the in vitro release results obtained indicated that all types of MSN provided a sustained release of the drug.

**Fig. 3:**
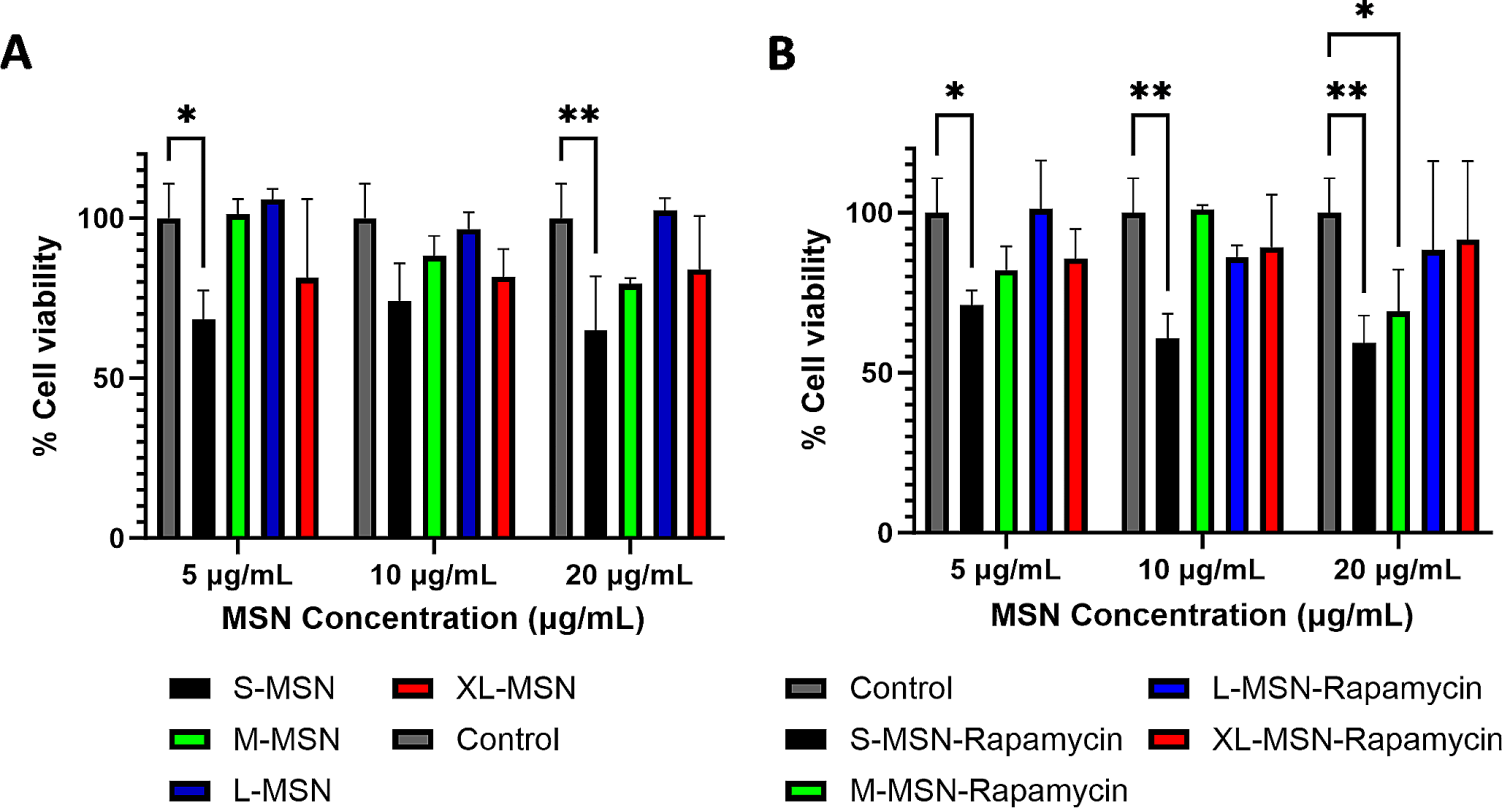
Cell viability results of DC2.4 cells incubated with non-loaded MSN **(A)** or rapamycin-loaded MSN **(B)** determined by WST-1 assay. Data are Mean ± SD, *n* = 3. Statistical analysis performed by Two-Way ANOVA, Dunnett correction for multiple comparisons. *p < 0.05; **p > 0.01

In order to test the biological behavior of the prepared rapamycin-loaded MSN, a series of in vitro experiments were carried out with a DC2.4 cells, a murine cell line that is widely used as a model for DCs. First, the cell viability of DC2.4 cells incubated with non-loaded or rapamycin-loaded MSN was evaluated. The results (Fig. 3) show that for non-loaded nanoparticles, only S-MSN presented a significant decrease in cell viability after incubation with the nanoparticles for 24 h at a concentration of 5 and 20 µg/mL (cell viability of 68.3 ± 9.14% and 65.02 ± 16.85%, respectively, Fig. 3A). No significant cell viability decrease was observed at 10 µg/mL for all non-loaded MSN treatment groups. A similar pattern was also observed for rapamycin-loaded MSN (Fig. 3B). S-MSN nanoparticles produced a significant reduction in DC2.4 cell viability at all tested concentrations, and M-MSN particles produced a significant reduction in cell viability only at the largest concentration (20 µg/mL). Rapamycin-loaded L-MSN and XL-MSN particles did not produce any significant effect in cell viability at any of the concentrations evaluated. Based on these results, 10 µg/mL was the concentration chosen for the experiments evaluating the biological effect of the nanoparticles.

**Fig. 4:**
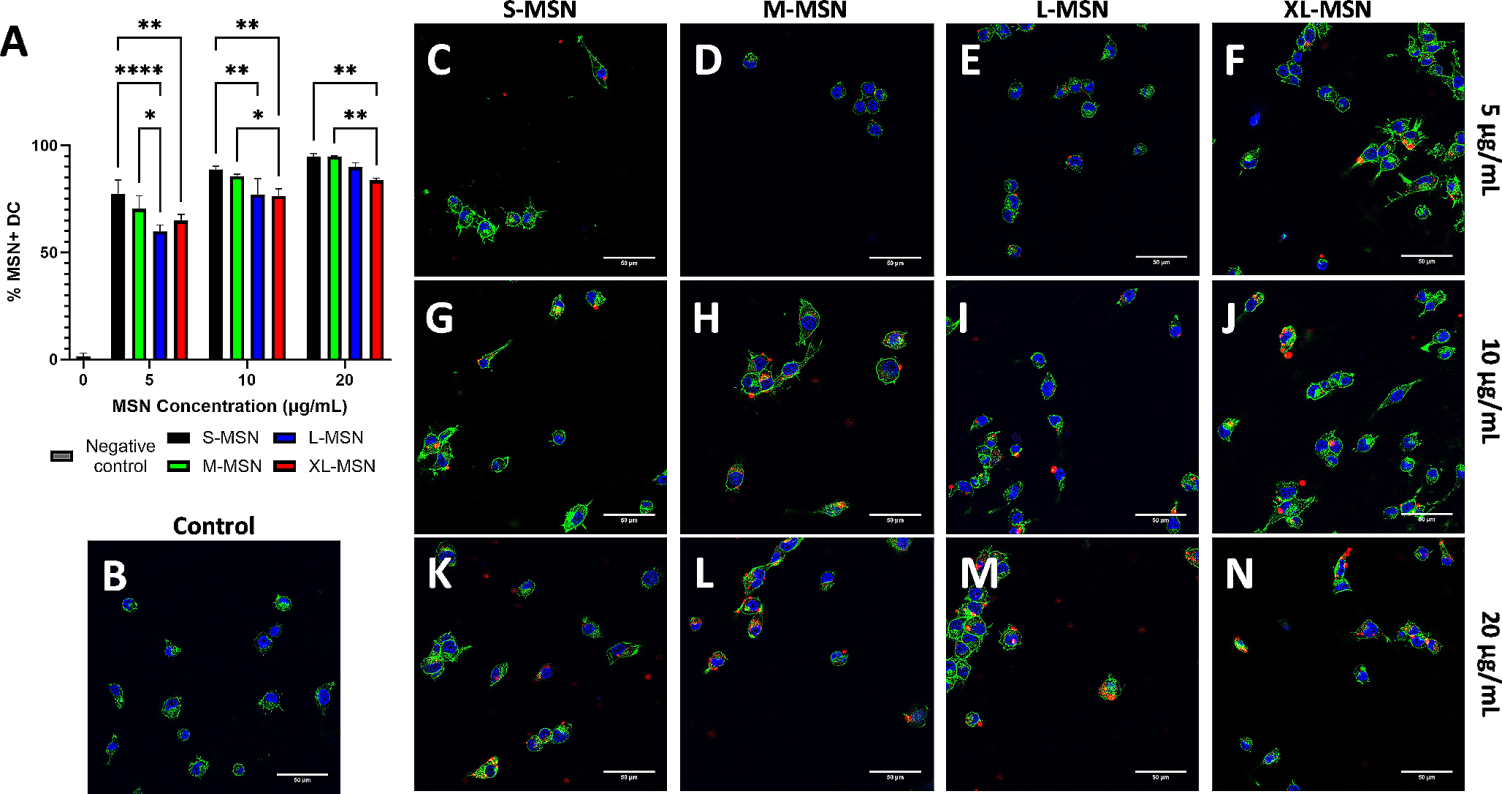
MSN uptake by DC2.4 determined by flow cytometry **(A)** and confocal microscopy **(B-N)**. Confocal microscopy images show DAPI-stained nuclei in blue, Phalloidin-Atto 488-stained cytoplasm in green and RITC-labeled MSN in red. Panels for control experiment without MSN **(B)**, and DC2.4 cells treated with S-MSN **(C,G,K)**, M-MSN **(D,H,L)**, L-MSN **(E,I,M)** or XL-MSN **(F,J,N)** at a dose of 5 **(C-F)**, 10 **(G-J)** or 20 **(K-N)** µg/mL. Data are Mean ± SD, *n* = 3. Statistical analysis performed by Two-Way ANOVA, Dunnett correction for multiple comparisons. *p < 0.05; **p > 0.01; ****p < 0.0001

To try to understand the interaction between the prepared MSN and DC2.4 cells, a nanoparticle uptake experiment was carried out using non-loaded RITC-labeled MSN, evaluating the results by flow cytometry and confocal microscopy (Fig. 4). Flow cytometry results showed that, as expected, nanoparticle uptake was largest for S-MSN particles and was reduced as mesopore size was increased. For each nanoparticle type, uptake showed to be dose-dependent, as the % of DC2.4 cells presenting nanoparticle fluorescence increased as the MSN dose became larger. These differences in nanoparticle uptake could be due to differences in nanoparticle surface roughness [40, 41], because of the differences in protein corona composition as a function of their pore sizes [39] or due to a combination of these factors. On the other hand, the larger nanoparticle uptake for S-MSN is likely related to the larger effect on cell viability observed in Fig. 3. Confocal microscopy images confirm the flow cytometry results and show MSN uptake for the different formulations at varying concentrations (Fig. 4B-N).

**Fig. 5:**
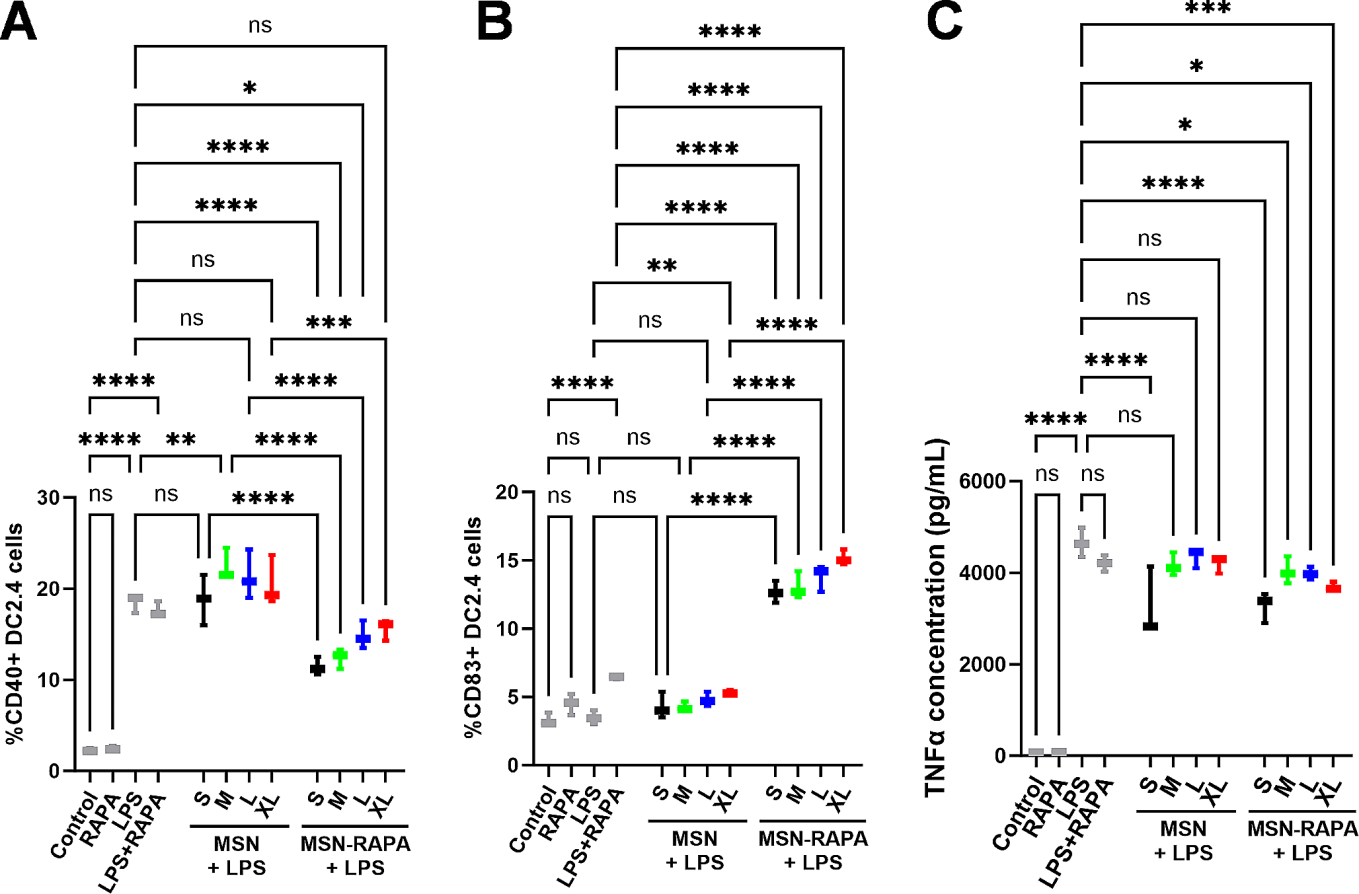
Evaluation of the expression of CD40 and CD83 in DC2.4 cells determined by flow cytometry after treatment with MSN at a nanoparticle concentration of 10 µg/mL **(A,B)**. Production of the pro-inflammatory cytokine TNF-α by DC2.4 cells determined by ELISA in culture medium supernatant after treatment with MSN at a nanoparticle concentration of 10 µg/mL **(C)**. Data are Mean ± SD, *n* = 3. Statistical analysis performed by Two-Way ANOVA, Dunnett correction for multiple comparisons. *p < 0.05; **p > 0.01; ***p < 0.001; ****p < 0.0001

Finally, the biological effect of non-loaded and rapamycin-loaded MSN on DC2.4 cells was evaluated by the expression of CD40, CD83 as DC activation markers and the production of the proinflammatory cytokine TNF-α. As rapamycin is an immunosuppressive drug, to test its pharmacological activity in vitro, an inflammatory stimulus (LPS) was also applied to the cells to evaluate if the drug-loaded nanoparticles could reverse its effect and compare the therapeutic effect of the different MSN formulations at a constant MSN concentration of 10 µg/mL (Fig. 5A-C). The results of CD40 expression (Fig. 5A) show that, as expected, treatment of DC2.4 cells with LPS produced a clear DC activation, with a drastic increase in CD40^+^ cells. Combination of LPS treatment with free rapamycin failed to inhibit this strong DC activation. On the other hand, neither free rapamycin alone nor treatment with non-loaded MSN or rapamycin-loaded MSN produced any significant increase in CD40 expression (Figure S2). When LPS-stimulated DC2.4 cells were also incubated with MSN, non-loaded MSN did not reduce CD40 expression (Fig. 5A), while rapamycin-loaded MSN inhibited of the effect of LPS in a pore-size dependent manner. Rapamycin-loaded S-MSN produced the largest decrease in CD40^+^ cells, with smaller inhibitory effect as particle pore became larger. In fact, treatment of LPS-stimulated cells with rapamycin-loaded XL-MSN did not produce any significant reduction in CD40 expression compared to LPS treatment alone. These results are coherent with the results of previous experiments, as S-MSN particles not only presented the largest rapamycin loading, but also the largest cellular uptake. It is worth noting that free rapamycin did not reduce CD40 expression, highlighting the importance of developing efficient nanocarriers of the drug to maximize its effect in the target immune cells. Next, we analyzed the effect of the different treatments on the expression of CD83 by DC2.4 cells. Although CD83 has also been traditionally considered as a DC activation marker, more recently it has been confirmed to play a role in immune tolerance generation, in a way that is still not fully understood. For example, Kryczanowsky et al., reported that tolerogenic DCs with high CD83 expression led to stronger induction of T regulatory responses than DCs with low CD83 expression [42]. In this regard, CD83 is currently not considered a typical co-stimulatory molecule, but rather a “master regulator in the development of adaptive immunity” [43]. In our experiment, while LPS stimulation did not change CD83 expression, there seemed to be a slight increase in free rapamycin-treated cells (which was then seen to not be statistically significant, Fig. 5B). Interestingly, the combination of LPS and free rapamycin did lead to a significant increase in CD83 expression (Fig. 5B). Non-loaded MSN did not induce any changes in CD83 expression (Figure S2), and in the combination of LPS + non-loaded MSN, only L-MSN and XL-MSN produced a slight significant increase in CD83^+^ cells compared to control (Figure S2). This might be consistent with previous reports that had shown some pore-size dependent effect of MSN on human DCs, which suggested their potential use as adjuvants to loaded antigenic molecules [20]. On the other hand, all rapamycin-loaded MSN produced a clear increase in CD83 expression, and the combination of LPS + all rapamycin-loaded MSN produced the largest CD83 expression. Although the multifaceted role that CD83 can play in the generation of different immunological responses might make it difficult to interpret these changes in isolation, the combination of a reduced CD40 expression with an increase in CD83 expression in LPS + rapamycin-loaded MSN (especially S-MSN and M-MSN) highlights the potential of the formulations here presented for immunomodulation. Finally, the production of the proinflammatory cytokine TNF-α (Fig. 5C) provides a very similar image to that obtained in the expression of CD40, with the same trends being observed, with all rapamycin-loaded MSN showing a significant reduction in TNF-α production in LPS-stimulated cells compared to LPS-only treatment. The only relevant difference in this case is the significant reduction in the production of TNF-α by the cells treated with non-loaded S-MSN, which might be due to the slight decrease in cell viability in this treatment group (Fig. 3), which would lead to decreased cytokine production just due to the smaller number of living cells in these wells. As the amount of rapamycin loaded within the different MSN types is different, it was possible that all the differences in MSN performance observed were due to the different dose of rapamycin present in each experimental group. To evaluate this, an equivalent experiment with DC2.4 cells was performed but maintaining a constant rapamycin concentration of 1 µg/mL. The results obtained (Figure S3) still show pore size-dependent trends similar to those described for the previous experiment regarding CD40 and CD83 expression as well as TNFα production. Furthermore, by maintaining rapamycin concentration constant, we could confirm that the formulation M-MSN indeed presented the best immunomodulatory potential, as even at constant cargo concentration, this formulation was the one that produced the largest inhibition of CD40 expression with the largest increase in CD83 expression without significantly affecting cell viability. Thus, this best-performing formulation could be further explored for therapeutic application as an immunomodulatory agent.

## Conclusions

In this work, MSN with 4 different pore sizes were prepared and characterized. Using these materials, pore size was shown to influence not only rapamycin loading and release, but also cytotoxicity, cellular uptake and the in vitro immunological response in a dendritic cell line model. By systematically evaluating the effect of the textural properties of rapamycin-loaded MSN on their biological performance, tailored MSN formulations can be designed with potential for precise immunomodulation for therapeutic application.

## Electronic supplementary material

Below is the link to the electronic supplementary material.

Supplementary Material 1

## Data Availability

The datasets generated during and/or analyzed during the current study are available from the corresponding author on reasonable request.
