# Correction: Immunomodulatory potential of rapamycin-loaded mesoporous silica nanoparticles: pore size-dependent drug loading, release, and in vitro cellular responses

**DOI:** 10.1007/s13346-024-01741-4

**Published:** 2024-11-13

**Authors:** Ana M. Pérez-Moreno, Carlos J. Aranda, María José Torres, Cristobalina Mayorga, Juan L. Paris

**Affiliations:** 1 Allergy Research Group, Instituto de Investigación Biomédica de Málaga y Plataforma en Nanomedicina- IBIMA Plataforma BIONAND. RICORS “Enfermedades inflamatorias”, Málaga, Spain; 2 Universidad de Málaga, Málaga, Spain; 3 Allergy Unit, Hospital Regional Universitario de Málaga-HRUM, Málaga, Spain; 4 Departamento de Medicina y Dermatología, Universidad de Málaga, Málaga, España

**Correction: Drug Delivery and Translational Research (2024) 14:3467–3476**

10.1007/s13346-024-01575-0

An additional affiliation was added for Ana M. Pérez-Moreno.

