# Supplementary material for: Immunomodulatory potential of rapamycin-loaded mesoporous silica nanoparticles: pore size-dependent drug loading, release, and in vitro cellular responses
Source: Drug Deliv Transl Res. 2024 Apr 1;14(12):3467–76. doi: 10.1007/s13346-024-01575-0 (PMC11499431; doi:10.1007/s13346-024-01575-0)
Supplement: Supplementary file 1 — Supplementary Material 1 [file 13346_2024_1575_MOESM1_ESM.pdf]

Supporting Information

Immunomodulatory Potential of Rapamycin-Loaded Mesoporous Silica Nanoparticles: Pore Size-Dependent Drug Loading, Release, and *In Vitro* Cellular Responses

Ana M. Pérez-Moreno[1], Carlos J. Aranda[1], María José Torres[1,2], Cristobalina Mayorga[1,2,*], Juan L Paris[1,*]

[1]Allergy Research Group, Instituto de Investigación Biomédica de Málaga y Plataforma en Nanomedicina-IBIMA Plataforma BIONAND. RICORS "Enfermedades inflamatorias", Málaga, Spain;

[2]Allergy Unit, Hospital Regional Universitario de Málaga-HRUM, Málaga, Spain

Corresponding authors e-mail: lina.mayorga@ibima.eu; juan.paris@ibima.eu

Table S1. Physicochemical characterization of the prepared MSN, including hydrodynamic diameter (D_H); Z potential; Brunauer, Emmett and Teller (BET) surface area; pore volume and pore width.

Material	Peak D_H (nm) Pristine MSN	Peak D_H (nm) Incubated with FBS	Z Potential (mV) Pristine MSN	Z Potential (mV) Incubated with FBS	BET Surface area (m^2/g)	Pore volume (cm^3/g)	Pore width (nm)
S-MSN	78.82	91.28	-4.09	-26.1	339.3	0.41	3.19
M-MSN	78.82	105.7	-6.94	-24.1	628.1	0.86	5.55
L-MSN	91.28	105.7	-13.7	-24.2	666.5	1.05	8.39
XL-MSN	91.28	122.4	-23.8	-24.5	695.3	1.34	11.3

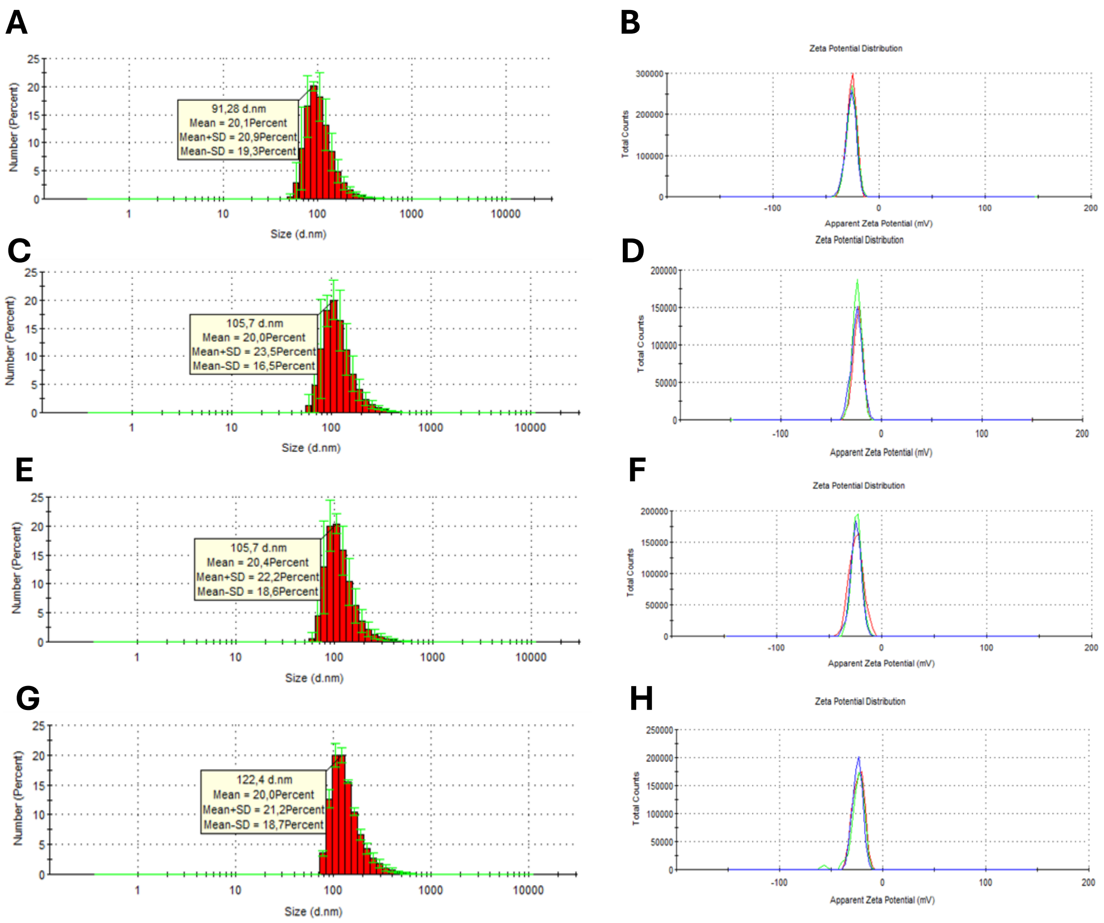

Figure S1. DLS (A,C,E,G) and Z potential (B,D,F,H) results from S-MSN (A,B), M-MSN (C,D), L-MSN (E,F) and XL-MSN (G,H) in water after incubation for 1 h in culture medium with 10 % FBS.

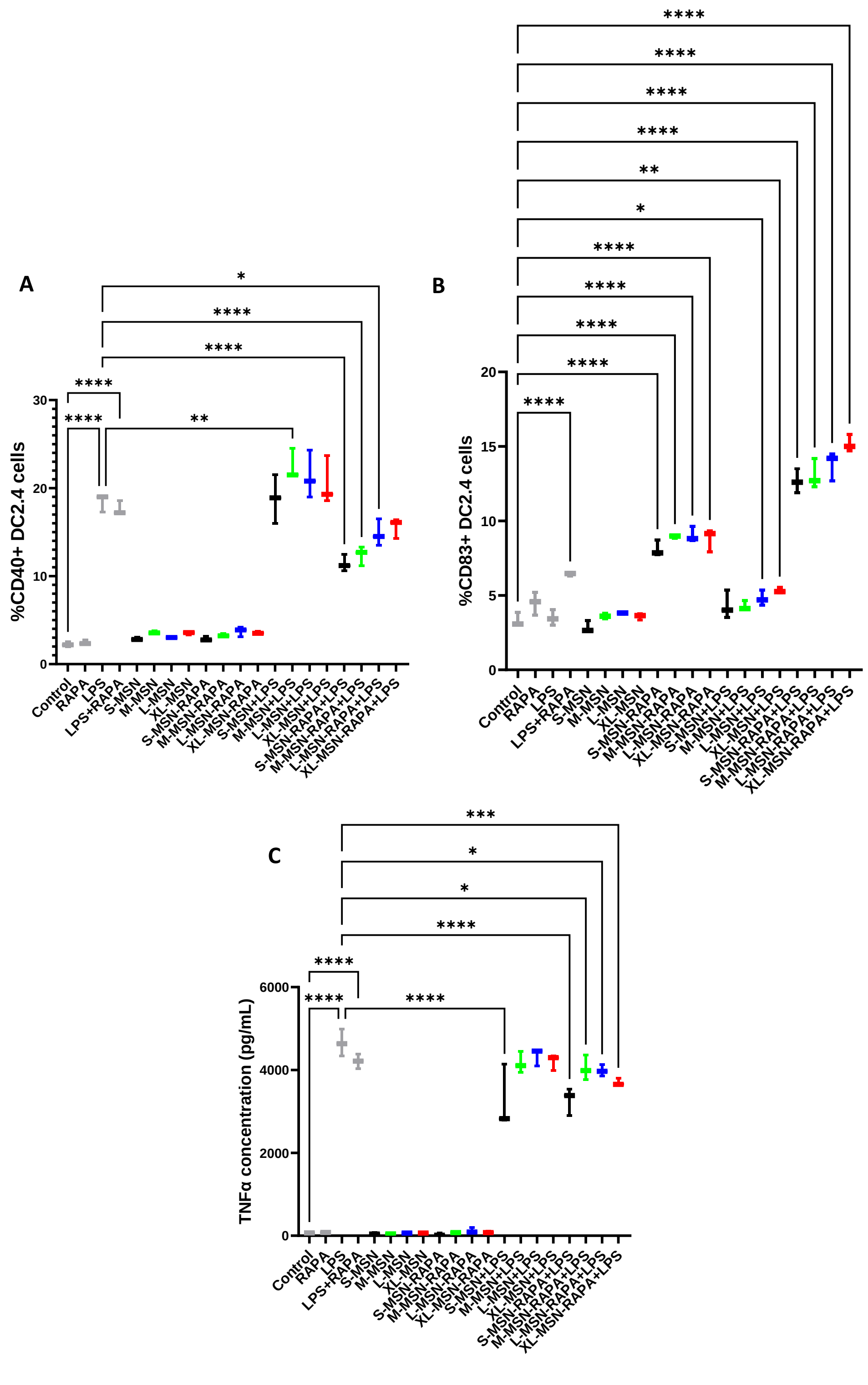

Figure S2. Evaluation of the expression of CD40 and CD83 in DC2.4 cells determined by flow cytometry after treatment with MSN at a nanoparticle concentration of 10 µg/mL (A,B). Production of the pro-inflammatory cytokine TNF-α by DC2.4 cells determined by ELISA in culture medium supernatant after treatment with MSN at a nanoparticle concentration of 10 µg/mL (C). Data are Mean ± SD, n=3. Statistical analysis performed by Two-Way ANOVA, Dunnett correction for multiple comparisons. *$p<0.05$; **$p>0.01$; ****$p<0.0001$.

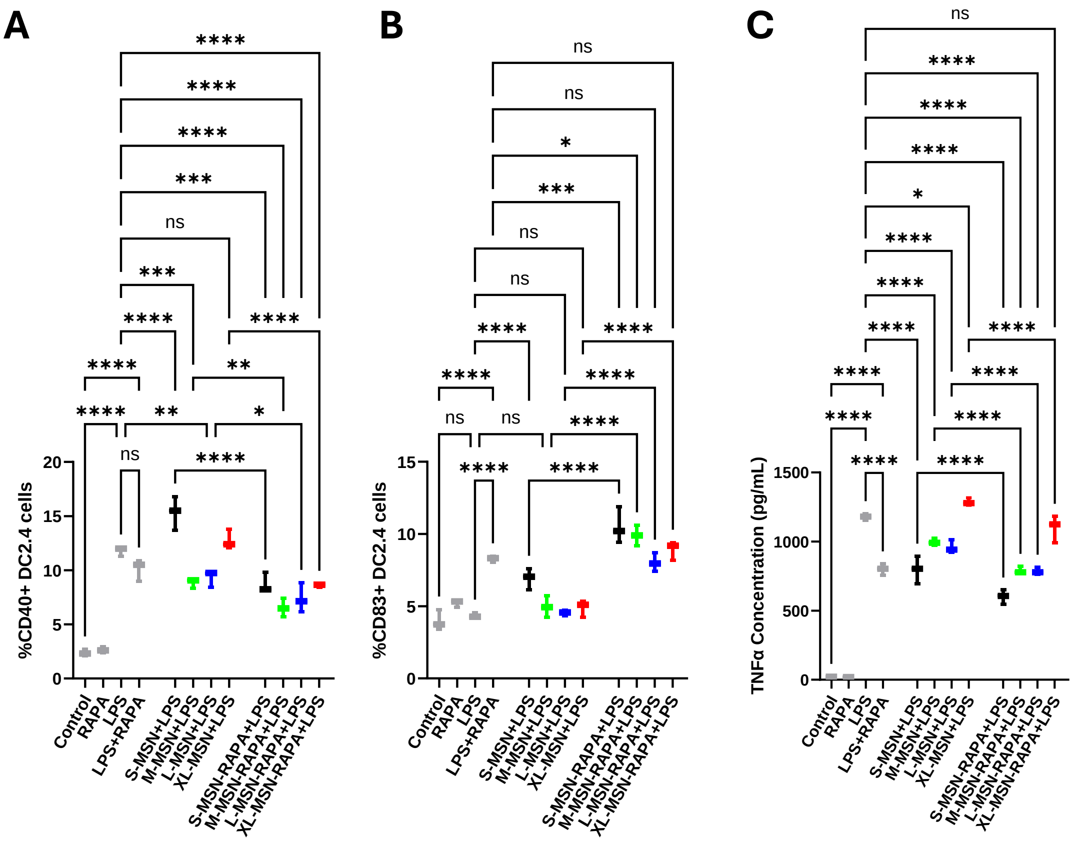

Figure S3. Evaluation of the expression of CD40 and CD83 in DC2.4 cells determined by flow cytometry after treatment with MSN at a rapamycin concentration of 1 µg/mL (or equivalent particle concentration for empty MSN) (A,B). Production of the pro-inflammatory cytokine TNF-α by DC2.4 cells determined by ELISA in culture medium supernatant after treatment with MSN at a rapamycin concentration of 1 µg/mL (or equivalent particle concentration for empty MSN) (C). Data are Mean ± SD, n=3. Statistical analysis performed by Two-Way ANOVA, Dunnett correction for multiple comparisons. *p<0.05; **p>0.01; ***p<0.001; ****p<0.0001.
